# Oaxaca-Blinder meets Kitagawa: What is the link?

**DOI:** 10.1371/journal.pone.0321874

**Published:** 2025-05-13

**Authors:** Ronald L. Oaxaca, Eva Sierminska

**Affiliations:** 1 Department of Economics, University of Arizona, Tucson, Arizona, United States of America; 2 IZA, Bonn, Germany; 3 GLO, Essen, Germany; 4 Luxembourg Institute of Socio-Economic Research (LISER), Esch/Alzette, Luxembourg; 5 INE PAN, Warsaw, Poland; 6 DIW, Berlin, Germany; University of Salamanca, SPAIN

## Abstract

Recently, papers have started combining the naming of two popular decomposition methods: the Oaxaca-Blinder (OB) method and the Kitagawa method, a popular method in demographics and sociology. Although the two approaches have the same objective in terms of decomposing outcome differences in some variable of interest between two populations, they are framed quite differently and do not overlap except in a special set of circumstances. In the light of Kitigawa’s early precedence, its more recent association with OB, and the seeming lack of any detailed analysis of the exact relationship between Kitigawa’s methodology in the literature, in this paper we show the precise relationship between Kitagawa’s approach and that of OB. The paper also provides a citation history of the papers.

## Introduction

In the 1950s, Evelyn M. Kitagawa, a professor of sociology and demography at the University of Chicago, published a paper “Components of a Difference between Two Rates" in the American Statistical Association Journal. The paper developed a method to decompose rates (or proportions) for two demographic groups by controlling for differences between these two groups in selected characteristics. This was done by applying the schedule of age-sex-race specific death rates for each of the groups to the age-sex-race composition of the standard population and then noting the difference between the two. One then can say with a certain confidence that the remaining differences after standardization are due to factors other than the age, sex and race composition. The method is referred to as “standardization" as the two rates are related to a standard population that has a specified age-sex-race composition.

Some 20 years later, Alan Blinder published a paper in 1973 in the Journal of Human Resources (JHR) using linear regression-based methods in which the mean difference in wages between two groups (a high-wage group and a low-wage group) is expressed as the sum of differentials attributable to differing endowments and those attributable to differing coefficients and an unexplained portion of the differential. In the same year, Ronald Oaxaca published a paper in the International Economic Review (IER) that estimates the average extent of discrimination faced by female workers in the United States. (Note: A conference volume version chapter was published the same year by Princeton University Press.) By interpreting the component corresponding to gender differences in coefficients as the Becker discrimination coefficient and using the properties of ordinary least squares estimation, Oaxaca shows that the wage differential between two groups can be expressed as the sum of the estimated effects of differences in individual characteristics and the estimated effects of discrimination. Although published in the same year, these two papers do not cite each other, although Blinder does thank Oaxaca in the footnote for helpful comments and cites the conference volume version. It is important to note that even though the two papers come from the same sub-field in Economics, the two authors were not aware of each others work nor of Kitagawa’s. This fact and the realization that in those times a low cross-fertilization across fields existed provides an indication of the reason Kitagawa (1955) had not been cited by either of the authors, although others such as Duncan (1968), for example - had been. For example, Blinder (1973) mentions on page 447 the contribution of Duncan and states that he used “a somewhat different specification and different statistical methods, but a decomposition technique which is almost the same as mine." Since their publication, these two papers have very often been cited together when referring to the Oaxaca-Blinder (OB) decomposition (see S1 Table in Supporting information). Based on Google Scholar, as of September 11, 2024, the Blinder paper has been cited 10,557 times and the Oaxaca paper 13,333 times. Recently, papers have started to combine the naming of the two methods referring to the Kitagawa-Oaxaca-Blinder (KOB) decomposition method or the pooled Kitagawa-Oaxaca-Blinder decomposition. As of September 11, 2024, we find 717 references to the KOB decomposition method in Google Scholar - over 600 of these occurring after 2020 (See S2 Table in Supporting information). This has caught our attention as no such canonical method exists and other decomposition methods have been developed since Kitagawa’s method in the 1950s. (For a review, see [4] and [3].) Why thus, the strong focus on the Kitagawa method?

We find that the largest increase in these “new” citations has been in Economics and the Social Sciences. As background, in the last decade the Economics profession has been attempting to undergo substantial changes. Numerous publications have highlighted the lack of diversity in the field and the differential treatment of women (See, for example, [14] for an overview.). Other attempts have been made to correct for the exclusion of women, for example, by providing a more complete view of economic history (see [2] and the edited volume.). We suspect that it is on this basis that there has been a renewed interest in the Kitagawa (1955) paper five decades later, as she has been rarely cited in Economics until 2020. None of this detracts from the originality and prescient contribution of Kitagawa’s path breaking work, which has been appreciated, popularized and extended in demography and sociology many decades before it became of interest in Economics. In this article, we do not focus on the issue of credit attribution, as described by [7] and referred to as the Mathew effect, which refers to the accrual of greater increments of recognition for particular scientific contributions to well-known scientists and the withholding of such recognition from scientists who have not yet made their mark. Nor, do we refer to the Matilda effect in Science, as introduced by [13] in reference to the forgotten women in Science. We do not argue for the existences of the Stigler’s law of eponomy in sociology of science, which says, “No scientific law is named after its original discoverer.” There [15] points out that in principle all science discoveries are multiples, which means they have usually been discovered previously by other individuals, but do not carry their name, as the naming convention needs to be accepted by the broader community. (In the paper, he shows a numerical example to confirm this on the basis of the normal (Gauss) distribution.) In fact, our main task is to identify in what capacity the two methods are identical or similar. We direct interested readers on the issue of credit attribution to the relevant cited literature.

As our investigation shows, although the two approaches have the same objective in terms of decomposing outcome differences in some variable of interest between two populations, they are framed quite differently and do not overlap except in a special set of circumstances.

This note establishes the conditions under which the two methodologies are identical. In the next section we focus on the legacy of the Kitagawa method. Then, we present an overview of each methodology, compare them, and in the following section offer a simple example of the set of circumstances in which they are identical. The final section summarizes the paper and concludes.

## The “Kitagawa" legacy and the recent surge in popularity

**Fig 1:**
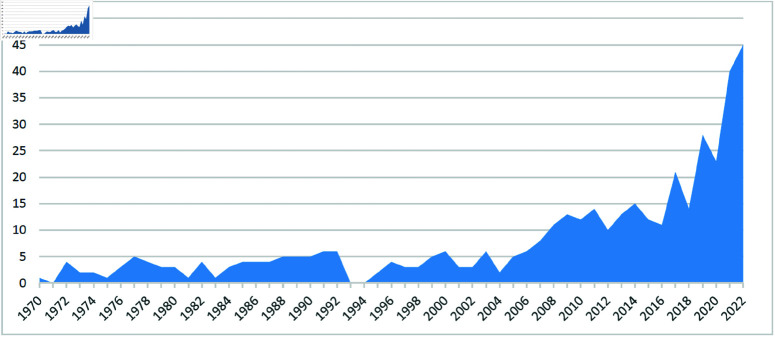
Citations of Kitagawa (1955) during the period 1970–2022.

The Kitagawa “standardization" method is a popular decomposition method in demography and sociology. It is discussed and extended in popular demographic textbooks including [12] and [3]. The Kitagawa (1955) paper has received over 1215 citations according to Google Scholar until September, 2024. (In the period 1954-1969 only 6 citations have been identified.) Scopus indicates that citations to Kitagawa’s seminal paper have been increasing annually over 1970–2022 (see Fig 1) and in particular over the last decade. As of March, 2023, there was a cumulative total of 415 citations (citations are not provided prior to 1970). According to the Web of Science, the 1955 paper has been cited 392 times - mostly in sociological and demographic journals, but more recently in other type of journals.

Table 1 shows the citation history by field, based on journals, the five previous decades and the last two years of this decade using the Web of Science. The results are striking. As in Fig 1 with Scopus, overall citations of the 1955 Kitagawa paper have doubled in the second decade of this century and in the last two years of this decade have reached those similar levels. In some fields, such as, Ecology and Statistics, the citation rates remains more or less unchanged. In Medicine and Public Health the increase is not as striking as in the social sciences. Here, the citation rates in 2010-19 at least doubled compared to the previous decade. In Sociology/Political Science it tripled and in Economics grew seven-fold. In the first two years of 2020, the citations in the social sciences have exceeded those of the previous decade. Thus, data suggests that the Kitagawa (1955) paper has gained in popularity - particularly more recently, but only in selected fields - in the social sciences - and in Economics more so than in any other field. As discussed previously this is most likely related to the changes taking place in the field itself.

**Table 1:** Citations of Kitagawa (1955) over time by fields.

Field	Decades						Total	Rate increase in citations	
	1970-79	1980-89	1990-99	2000-09	2010-19	2020-23		2010-19	2020-23
Ecology/Geography	1	2	1	0	3	5	12		
Economics	0	2	0	1	7	17	27	7	2.4
Population/Demography	12	10	10	18	29	28	107	2	1.0
Social Science	8	3	3	8	13	23	58	2	1.8
Sociology/ Political Science	12	11	3	4	12	16	58	3	1.3
Medicine	0	12	8	11	27	10	68	2	0.4
Public Health	1	4	0	12	16	3	36	1	0.2
Statistics/ Quantative Methods	1	4	4	0	4	1	14		0.3
Other (e.g. Law, Science, Psychology)	0	0	3	2	4	3	12	2	0.8
Total	35	48	32	56	115	106	392		

*Source:* Web of Science (March, 2023)

*Notes:* Calculation based on journal classification available from authors upon request.

## The two decomposition methods: Is there a link?

### The Kitagawa decomposition

Kitagawa never explicitly referred to her method as a decomposition, though it clearly was a decomposition method. Nor did she frame her method in terms of linear regressions. Kitagawa’s decomposition technique is closely related to standardization methods that address such questions as “How much of the difference between death rates in A and B is attributable to differences in their age (or race and sex) distributions?" The development of Kitagawa’s method was based on previous standardization methods used in sociology and demography, which identified for example, “influences of changes in age distribution" and “influences of changes in occurrence rates" and are included in [5] Handbook of Statistical Methods for Demographers on standardization methods. The main contribution of Kitagawa was to develop a technique called “components of a difference between two rates." The goal of this technique was to explain the difference between rates of two groups by decomposing the difference into differences in their specific rates and differences in their composition. This is the two-component method. Kitagawa also develops a three-component method in which the group difference in rates is decomposed into differences in their specific rates, differences in their composition, and differences attributable to the interaction between group differences in their specific rates and group differences in their composition.

The Kitagawa methodology is broader than the prior techniques for standardization rates, which were developed to summarize and compare differences in two (or more) sets of specific rates and can provide counterfactual outcome rates based on assumed standard variables.

### The Oaxaca-Blinder decomposition

Unlike the original Kitagawa method, the OB decomposition is framed in terms of linear regression methods applied to the mean difference between two groups in some outcome variable. This mean group outcome difference is decomposed into the differences arising from group differences in the parameters and group differences in characteristics. The OB decomposition methodology can also include a term that captures the interaction between group parameter differences and group characteristics. Although the method has been significantly extended to decompositions associated with quantile regression, limited dependent variables, etc., these extensions still fit under the OB heading (See [4] for more details.), as they separate group differences into group differences coming from characteristics and others.

### Kitagawa vs OB: A comparison of the two methods

Our investigation in the following section, reveals that the Kitagawa decomposition method is a special case of OB, or equivalently because of Kitagawa precedence, OB is a generalization of the Kitagawa decomposition method. In particular, the OB methodology is identical/equivalent to the Kitagawa methodology when the dependent outcome variables are binary, the covariates are categorical variables comprised of sets of indicator variables, and the OB decomposition uses the OLS estimated linear probability model (LPM). In short, all Kitagawa decompositions are OB, but not all OB are Kitagawa decompositions. A detailed example of the conditions described above is presented in Appendix S1.

An interesting aspect of the Kitagawa methodology is that a researcher does not require the underlying micro data set to conduct the decompositions, only the requisite proportions/rates. The regression framework analog is that the researcher would require only the requisite sample moments. Such circumstances might arise if there are privacy concerns about identifying individuals. Disclosing only the sample proportions or sample moments would permit the researcher to conduct the decompositions without breaching confidentiality.

## Exploring the link between the two methods: an illustrative example

We illustrate the relationship between Kitagawa’s decomposition methodology and the OB approach by examining Kitagawa’s method in terms of one factor (explanatory variable) and the two-component decomposition ([6], p. 1182). The derivation details for the illustrative example can be found in S1 Appendix. With some notational changes from that used by Kitagawa, suppose there are two population groups, *A* and *B*, for which the group mean difference in a binary indicator outcome variable *Y* is decomposed. The sample sizes for the two population groups are denoted by ${\text{N}}^{\text{A}}$ and ${\text{N}}^{\text{B}}$. The explanatory variable *X* is a categorical variable defined by a mutually exclusive set of *K* indicator variables such that $\sum_{k=1}^{K}X_{ik}=1\;\forall i$ in a group sample, where *i* refers to the *i*th individual. Accordingly, the group outcome proportion difference to be decomposed is given by


$${\overset{―}{Y}}^{A}-{\overset{―}{Y}}^{B}=\frac{\sum_{i=1}^{N^{A}}Y_{i}^{A}}{N^{A}}-\frac{\sum_{i=1}^{N^{B}}Y_{i}^{B}}{N^{B}}$$

$$=\frac{N_{Y}^{A}}{N^{A}}-\frac{N_{Y}^{B}}{N^{B}},$$ (1)

where $Y_{i}^{A}$, $Y_{i}^{B}$ are the binary outcome indicators for the *i*th individuals in groups *A* and *B*, and $N_{Y}^{A},N_{Y}^{B}$ are the numbers of group *A* and *B* individuals for whom *Y*_*i*_ = 1.

Kitagawa’s one-factor, two-component decomposition is given by

$${\overset{―}{Y}}^{A}-{\overset{―}{Y}}^{B}=\text{Gross}\;X+\text{Residual}\;X,$$ (2)

where

$$\text{Gross}\;X=\sum_{k=1}^{K}\left(\frac{{\overset{―}{Y}}_{k}^{A}+{\overset{―}{Y}}_{k}^{B}}{2}\right)\left(\frac{N_{k}^{A}}{N^{A}}-\frac{N_{k}^{B}}{N^{B}}\right)$$ (3)

$$\text{Residual}\;X=\sum_{k=1}^{K}\frac{\left(\frac{N_{k}^{A}}{N^{A}}+\frac{N_{k}^{B}}{N^{B}}\right)}{2}\left({\overset{―}{Y}}_{k}^{A}-{\overset{―}{Y}}_{k}^{B}\right).$$ (4)

Note that the group weights used to provide the decomposition reference group in Eq 3 and Eq 4 are the simple averages of the *Y* outcome weights and the *X*-specific compositional rates. Gross *X* measures how much of the group difference in the mean outcome arises from group compositional differences in the explanatory variable *X*. The Residual *X* component measures how much of the group difference in the mean outcome arises from group differences in the *X*-specific *Y* outcome rates.

As explained in the Appendix, ${\overset{―}{Y}}_{k}^{A}$ and ${\overset{―}{Y}}_{k}^{B}$ denote the outcome rates among individuals in the *k*th category of variable *X* for groups *A* and *B* (for k=1,...,K):


$${\overset{―}{Y}}_{k}^{A}=\frac{\sum_{i=1}^{N^{A}}Y_{i}^{A}X_{ik}}{N_{k}^{A}}=\frac{N_{Yk}^{A}}{N_{k}^{A}}$$

$${\overset{―}{Y}}_{k}^{B}=\frac{\sum_{i=1}^{N^{B}}Y_{i}^{B}X_{ik}}{N_{k}^{B}}=\frac{N_{Yk}^{B}}{N_{k}^{B}},$$

where $N_{Yk}^{A}=\sum_{i=1}^{N^{A}}Y_{i}^{A}X_{ik}$ and $N_{Yk}^{B}=\sum_{i=1}^{N^{B}}Y_{i}^{B}X_{ik}$ represent the number of individuals in groups *A* and *B* for whom $(Y_{i}^{A}\cdot X_{ik})=1$ and $(Y_{i}^{B}\cdot X_{ik})=1$, and $N_{k}^{A}$ and $N_{k}^{b}$ represent the numbers of individuals in each group for whom *X*_*k*_ = 1. Thus, $N_{Yk}^{A}$ and $N_{Yk}^{B}$ are the numbers of individuals in the *X* specific category for whom the outcome variable  = 1, and $\left(\frac{N_{k}^{A}}{N^{A}}\right)$ and $\left(\frac{N_{k}^{B}}{N^{B}}\right)$ are the *X* specific compositional weights.

Next, we examine the OB decomposition [1, 9]. With the same data set, the OB regression-based approach would specify LPMs for the two population groups:

$$Y_{i}^{A}=\sum_{k=1}^{K}X_{ik}\beta_{k}^{A}+\epsilon_{i},\;i=1,...,N^{A}$$ (5)

$$Y_{i}^{B}=\sum_{k=1}^{K}X_{ik}\beta_{k}^{B}+\epsilon_{i},\;i=1,...,N^{B}.$$

Note that there is no separate constant term as the indicator variables sum to 1.

Typically, one counterfactually assigns one of the two population groups to be the reference group. In the present case, we follow Kitagawa’s counterfactual and adopt the simple average of the two groups’s estimated coefficients, which turn out to be identical to each group’s K-specific rates seen in the Kitagawa decomposition:


$$b_{k}^{*}=\frac{\left(b_{k}^{A}+b_{k}^{B}\right)}{2},\;k=1,...,K$$

$$=\frac{\left({\overset{―}{Y}}_{k}^{A}+{\overset{―}{Y}}_{k}^{B}\right)}{2}.$$ (6)

Accordingly, the OLS decomposition is given by


$${\overset{―}{Y}}^{A}-{\overset{―}{Y}}^{B}=\sum_{k=1}^{K}{\overset{―}{X}}_{k}^{A}b_{k}^{A}-\sum_{k=1}^{K}{\overset{―}{X}}_{k}^{B}b_{k}^{B}$$

$$=\underset{\text{Explained}}{\underbrace{\sum_{k=1}^{K}\left({\overset{―}{X}}_{k}^{A}-{\overset{―}{X}}_{k}^{B}\right)b_{k}^{*}}}+\underset{\text{Unexplained}}{\underbrace{\sum_{k=1}^{K}{\overset{―}{X}}_{k}^{A}\left(b_{k}^{A}-b_{k}^{*}\right)+\sum_{k=1}^{K}{\overset{―}{X}}_{k}^{B}\left(b_{k}^{*}-b_{k}^{B}\right)}}.$$ (7)

In the Appendix, we show that the Kitagawa decomposition components in this example are identical to the OB decomposition components:

$$\text{Gross}\;X=\sum_{k=1}^{K}\left(\frac{{\overset{―}{Y}}_{k}^{A}+{\overset{―}{Y}}_{k}^{B}}{2}\right)\left(\frac{N_{k}^{A}}{N^{A}}-\frac{N_{k}^{B}}{N^{B}}\right)=\underset{\text{Explained}}{\underbrace{\sum_{k=1}^{K}\left({\overset{―}{X}}_{k}^{A}-{\overset{―}{X}}_{k}^{B}\right)b_{k}^{*}}}$$ (8)

$$\text{Residual}\;X=\sum_{k=1}^{K}\frac{\left(\frac{N_{k}^{A}}{N^{A}}+\frac{N_{k}^{B}}{N^{B}}\right)}{2}\left({\overset{―}{Y}}_{k}^{A}-{\overset{―}{Y}}_{k}^{B}\right)=\underset{\text{Unexplained}}{\underbrace{\sum_{k=1}^{K}{\overset{―}{X}}_{k}^{A}\left(b_{k}^{A}-b_{k}^{*}\right)+\sum_{k=1}^{K}{\overset{―}{X}}_{k}^{B}\left(b_{k}^{*}-b_{k}^{B}\right)}}.$$ (9)

The counterfactual assumption in the above example is a special case of the generalized decomposition described in [8, 11] and [10]. If one adopts either of the group’s *X*-specific rates as the standard (reference) outcome rate, the Kitagawa decomposition would correspond exactly to the OB decomposition in which the same group’s estimated coefficients are used for the counterfactual.

## Conclusion

To our knowledge, our paper is the first explicit recognition of the exact relationship between the Kitagawa decomposition and OB decomposition methodologies. The two methodologies are identical only in the very specific set of circumstances characterized by binary dependent outcome variables, all covariates are categorical variables comprised of sets of mutually exclusive indicator variables, and the OB decomposition uses OLS estimated LPMs. As the OB and OB-related methodologies do not require the restrictions on the dependent and independent variables inherent in the original Kitagawa methodology, the OB decomposition methodology is more general. Kitagawa did not recognize her method as being regression based and to our knowledge this has not been previously shown. Another challenge is that the regression equivalence of Kitagawa’s method is limited to the data restrictions unique to her approach. Without recognition of this point, it might erroneously appear that the original Kitagawa method can be readily applied to data sets that do not satisfy the mentioned data restrictions.

The cross-fertilization of fields, in particular with that of economics, has given a new wave of popularity to the Kitagawa name. Yet, in our opinion, it would not give the original Kitagawa method justice if it were cited unknowingly In this paper, as far as we know, we show for the first time what is the relationship between the two methodologies.

For more information, see Supporting information.

## Supporting information

S1 Appendix Example of the Kitagawa and Oaxaca-Blinder decomposition. (PDF)

S1 Table Paper citations. (PDF)

S2 Table Citations of Oaxaca-Blinder and Kitagawa-Oaxaca-Blinder methods. (PDF)
