# Supplementary material for: Oaxaca-Blinder meets Kitagawa: What is the link?
Source: PLoS One. 2025 May 13;20(5):e0321874. doi: 10.1371/journal.pone.0321874 (PMC12074319; doi:10.1371/journal.pone.0321874)
Supplement: S1 Appendix — (PDF) [file pone.0321874.s001.pdf]

Oaxaca-Blinder meets Kitagawa: What is the link?

Supporting information

S1 Appendix. Example of the Kitagawa and Oaxaca-Blinder decomposition This appendix provides a detailed example of the conditions under which the Kitagawa and Oaxaca-Blinder decompositions are equivalent. The derivations below satisfy the data restrictions of the Kitagawa decomposition. At the level of the individual, the Kitagawa data restrictions are that the outcome variable of interest is an indicator variable, and the covariates are categorical variables comprised of sets of mutually exclusive indicator variables that sum to 1. We show that the Kitagawa decomposition is equivalent to the OB decomposition based on OLS-estimated linear probability models estimated with the same data.

Our example is a one factor (explanatory variable), two-component decomposition (Kitagawa 1955 p.1182). With some notational changes from the original Kitagawa paper, suppose there are two population groups, A and B, for which the group mean difference in a binary indicator outcome variable Y is to be decomposed (e.g. 0/1 not employed/ employed). The sample sizes for the two population groups are denoted by N^A and N^B. The one-factor, explanatory variable X is a categorical variable defined by a mutually exclusive set of K indicator variables such that $\sum_{k=1}^{K} X_{ik} = 1 \; \forall i$ in a group sample, where i refers to the ith individual (e.g. educational groups: no high school/ high school/ more than high school). Let Y_i^A and Y_i^B denote the outcome indicator variable for the ith individual in groups A and B.

The group mean proportions are given by

$$\overline{Y}^A = \frac{\sum_{i=1}^{N^A} Y_i^A}{N^A} = \frac{N_Y^A}{N^A}$$

$$\overline{Y}^B = \frac{\sum_{i=1}^{N^B} Y_i^B}{N^B} = \frac{N_Y^B}{N^B},$$

where N_Y^A, N_Y^B are the numbers of individuals in groups A and B for whom $Y_i = 1$. Accordingly, the group mean outcome proportion to be decomposed is given by

$$\overline{Y}^A - \overline{Y}^B = \frac{N_Y^A}{N^A} - \frac{N_Y^B}{N^B}.$$

Next, let $\overline{Y}_k^A$ and $\overline{Y}_k^B$ denote the outcome rates among individuals in the kth category of variable X for groups A and B:

$$\begin{aligned} \overline{Y}_k^A &= \frac{\sum_{i=1}^{N^A} Y_i^A X_{ik}}{N_k^A},\; k = 1, ..., K \\ &= \frac{N_{Yk}^A}{N_k^A} \end{aligned} \tag{1}$$

$$\overline{Y}_k^B = \frac{\sum_{i=1}^{N^B} Y_i^B X_{ik}}{N_k^B},\ k = 1, ..., K$$
$$= \frac{N_{Yk}^B}{N_k^B}, \tag{2}$$

where $N_{Yk}^A = \sum_{i=1}^{N^A} Y_i^A X_{ik}$ and $N_{Yk}^B = \sum_{i=1}^{N^B} Y_i^B X_{ik}$ represent the number of individuals in groups A and B for whom $(Y_i^A \cdot X_{ik}) = 1$ and $(Y_i^B \cdot X_{ik}) = 1$, or in other words, N_{Yk}^A and N_{Yk}^B are the number of individuals who fall in the kth indicator category and for whom the Y outcome indicator variable = 1. Now let N_k^A, N_k^b represent the numbers of individuals in each group for whom $X_k = 1$. Kitagawa shows that the overall outcome rate for each population group is equal to the sum of its own X specific outcome rates weighted by its own X composition $\left(\frac{N_k^A}{N^A}\right), \left(\frac{N_k^B}{N^B}\right)$:

$$\overline{Y}^A = \sum_{k=1}^{K} (\overline{Y}_k^A) \left(\frac{N_k^A}{N^A}\right)$$

$$\overline{Y}^B = \sum_{k=1}^{K} (\overline{Y}_k^B) \left(\frac{N_k^B}{N^B}\right).$$

Thus,

$$\overline{Y}^A - \overline{Y}^B = \sum_{k=1}^{K} (\overline{Y}_k^A) \left(\frac{N_k^A}{N^A}\right) - \sum_{k=1}^{K} (\overline{Y}_k^B) \left(\frac{N_k^B}{N^B}\right).$$

Kitagawa's two-component decomposition of the group difference in outcome rates consists of (1) a Gross X component which is that portion of the group outcome rate difference attributable to group differences in their X composition $\left(\frac{N_k^A}{N^A} - \frac{N_k^B}{N^B}\right)$, and (2) a Residual X component which is that portion of the group outcome rate difference attributable to group differences in their X specific outcome rates $\left(\frac{\overline{Y}_k^A + \overline{Y}_k^B}{2}\right)$ (Kitagawa 1955 p.1182):

$$\overline{Y}^A - \overline{Y}^B = \text{Gross } X + \text{Residual } X,$$

$$= \underbrace{\sum_{k=1}^{K} \left(\frac{\overline{Y}_k^A + \overline{Y}_k^B}{2}\right) \left(\frac{N_k^A}{N^A} - \frac{N_k^B}{N^B}\right)}_{\text{Gross X}} + \underbrace{\sum_{k=1}^{K} \frac{\left(\frac{N_k^A}{N^A} + \frac{N_k^B}{N^B}\right)}{2} \left(\overline{Y}_k^A - \overline{Y}_k^B\right)}_{\text{Residual X}}.$$

Note that the average of each group's X specific rates is used as the standard (reference) group outcome rate.

We now examine the OB regression-based decomposition. With the same data, the regression-based approach would specify linear probability models for the two

population groups:

$$Y_i^A = \sum_{k=1}^{K} X_{ik}\beta_k^A + \epsilon_i,\ i = 1, ..., N^A$$

$$Y_i^B = \sum_{k=1}^{K} X_{ik}\beta_k^B + \epsilon_i,\ i = 1, ..., N^B.$$

Note that there is no separate constant term as the indicator variables sum to 1.

Because the indicator variables comprising X are mutually exclusive, the cross-product matrix for the OLS estimator of the β's is a diagonal matrix. This orthogonal design means that the OLS estimator simply corresponds to the separate simple regressions of Y_i on each X_{ik} indicator variable:

$$b_k^A = \frac{\sum_{i=1}^{N^A} X_{ik} Y_i^A}{\sum_{i=1}^{N^A} X_{ik}^2},\ k = 1, ..., K \tag{3}$$

$$= \frac{N_{Yk}^A}{N_k^A} = \overline{Y}_k^A.$$

$$b_k^B = \frac{\sum_{i=1}^{N^B} X_{ik} Y_i^B}{\sum_{i=1}^{N^B} X_{ik}^2},\ k = 1, ..., K \tag{4}$$

$$= \frac{N_{Yk}^B}{N_k^B} = \overline{Y}_k^B.$$

We see that the OLS coefficient estimates correspond to Kitagawa's X specific outcome rates derived in eq(1) and eq(2). Furthermore, the sample indicator variable means correspond to the Kitagawa X compositions:

$$\overline{X}_k^A = \frac{\sum_{i=1}^{N^A} X_{ik}}{N^A},\ k = 1, ..., K$$

$$= \frac{N_k^A}{N^A}$$

$$\overline{X}_k^B = \frac{\sum_{i=1}^{N^B} X_{ik}}{N^B},\ k = 1, ..., K$$

$$= \frac{N_k^B}{N^B}.$$

Typically, one group is assigned as the reference group in the standard (OB) OLS decomposition. Here, we follow Kitagawa's counterfactual and adopt simple averages of the two groups's estimated coefficients, which is the same as the average of each group's

K - specific rates seen in the Kitagawa decomposition:

$$b_k^* = \frac{(b_k^A + b_k^B)}{2},\ k = 1, ..., K$$
$$= \frac{(\overline{Y}_k^A + \overline{Y}_k^B)}{2}.$$

Accordingly, the OLS decomposition is as follows:

$$\overline{Y}^A - \overline{Y}^B = \sum_{k=1}^{K} \overline{X}_k^A b_k^A - \sum_{k=1}^{K} \overline{X}_k^B b_k^B$$

$$= \underbrace{\sum_{k=1}^{K} \left(\overline{X}_k^A - \overline{X}_k^B\right) b_k^*}_{\text{Explained}} + \underbrace{\sum_{k=1}^{K} \overline{X}_k^A \left(b_k^A - b_k^*\right) + \sum_{k=1}^{K} \overline{X}_k^B \left(b_k^* - b_k^B\right)}_{\text{Unexplained}}.$$

Note that simple algebra establishes the following relationship among the LPM coefficients: $\frac{b_k^A - b_k^B}{2} = b_k^A - b_k^* = b_k^* - b_k^B$. We now show with simple subsitution that the Kitagawa decomposition components correspond exactly to the OB decomposition components:

$$\underbrace{\sum_{k=1}^{K} \left(\frac{\overline{Y}_k^A + \overline{Y}_k^B}{2}\right) \left(\frac{N_k^A}{N^A} - \frac{N_k^B}{N^B}\right)}_{\text{Gross X}} = b_k^* \sum_{k=1}^{K} \left(\overline{X}_k^A - \overline{X}_k^B\right)$$

$$= \underbrace{\sum_{k=1}^{K} \left(\overline{X}_k^A - \overline{X}_k^B\right) b_k^*}_{\text{Explained}}$$

$$\underbrace{\sum_{k=1}^{K} \frac{\left(\frac{N_k^A}{N^A} + \frac{N_k^B}{N^B}\right)}{2} \left(\overline{Y}_k^A - \overline{Y}_k^B\right)}_{\text{Residual X}} = \sum_{k=1}^{K} \left(\frac{\overline{X}_k^A + \overline{X}_k^B}{2}\right) \left(b_k^A - b_k^B\right)$$

$$= \sum_{k=1}^{K} \overline{X}_k^A \left(\frac{b_k^A - b_k^B}{2}\right) + \sum_{k=1}^{K} \overline{X}_k^B \left(\frac{b_k^A - b_k^B}{2}\right)$$

$$= \underbrace{\sum_{k=1}^{K} \overline{X}_k^A \left(b_k^A - b_k^*\right) + \sum_{k=1}^{K} \overline{X}_k^B \left(b_k^* - b_k^B\right)}_{\text{Unexplained}}.$$

Thus, Kitagawa's Gross K component portion of the group outcome rate difference attributable to group differences in their X composition is identical to the explained difference in the OB decomposition, and Kitagawa's Residual X component portion of

the group outcome rate difference attributable to group differences in their X specific outcome rates is identical to the unexplained difference in the OB decomposition.

Under the special conditions shown above, it is straightforward to show that the equivalence between the Oaxaca-Blinder and Kitagawa decompositions holds whether the counterfactual standard corresponds to one of the two groups or is a weighted average.

S1 Table. Paper citations.

Source: Google Scholar (Sept. 11, 2024)

	Paper		
**Time**	**Kitagawa (1955)**	**Oaxaca (1973)**	**Blinder (1973)**
Overall	1 215	13 333	10 557
Since 2024	112	487	441
Since 2023	234	1 190	1 090
Since 2020	465	3 290	2 960
2011-2020	345	6 150	5 130
1991-2010	262	4 210	2 680
1970-1990	162	343	334

S2 Table. Citations of Oaxaca-Blinder and Kitagawa-Oaxaca-Blinder methods.

Source: Google Scholar (Sept. 11, 2024)
Notes: The order of keywords has no impact on the search outcome.

	Keywords	
**Time**	**Oaxaca Blinder**	**Kitagawa Oaxaca Blinder**
Since 1970	16 300	717
Since 2024	1 240	162
Since 2023	3 180	339
Since 2020	8 180	611
2011-2020	11 700	107
1991-2010	4 470	26
1970-1990	270	7
